# Hailey-Hailey Disease with Superimposed Eczema Herpeticum Caused by Herpes Simplex Virus Type 2 Infection in a Burn Unit: A Case Report and Literature Review

**DOI:** 10.7759/cureus.5907

**Published:** 2019-10-14

**Authors:** An Guo Michael Chin, Mohammed Asif, Charles Hultman, Julie Caffrey

**Affiliations:** 1 General Surgery, St. John's Episcopal Hospital, Far Rockaway, USA; 2 Surgery, Burn Center, The Johns Hopkins University School of Medicine, Baltimore, USA; 3 Plastic Surgery, The Johns Hopkins University School of Medicine, Baltimore, USA

**Keywords:** hailey-hailey disease, familial benign pemphigus, eczema herpeticum, kaposi varicelliform, herpes simplex virus type 2, burn, herpetic eczema, low dose naltrexone

## Abstract

Familial benign pemphigus, or Hailey-Hailey disease (HHD), is a rare (1 in 50,000), benign, autosomal dominant cutaneous disorder that causes a painful rash and blistering commonly occurring in the intertriginous folds. Despite having a good prognosis, there is no cure for HHD and the disease can be quite debilitating to the quality of life. The complexity of HHD can be compounded by superimposed eczema herpeticum (EH) or Kaposi's varicelliform eruption, which is caused by a viral infection occurring in preexistent cutaneous conditions. We present a unique clinical presentation of HHD with superimposed EH caused by herpes simplex virus type 2 (HSV-2) infection managed in a burn unit. It is highly advised that a recalcitrant HHD with superimposed EH caused by HSV-2 infection should be managed in burn centers that offer multimodalities for prompt, rigorous management. Early diagnosis and treatment are highly suggested for EH to avoid fatal complications.

## Introduction

Familial benign pemphigus, or Hailey-Hailey disease (HHD) (IMIM#16900), was first described in 1939 [1-2]. It is a rare (1 in 50,000), benign, autosomal dominant cutaneous disorder that causes a painful rash and blistering commonly occurring in intertriginous folds [3]. Although there is no present cure, HHD has a good prognosis. It is caused by a mutation in the plasma membrane-related Ca 2+ -ATPase-1 (ATP2C1) gene at 3q22, which is responsible for constructing the human secretory pathway Ca 2+ / Mn 2 ± ATPase protein 1 (hSPCA1) protein or adenosine triphosphate calcium pump located in the Golgi apparatus [4]; hSPCA1 helps regulate calcium storage, which plays an essential role for cellular activities, such as cell growth and cell adhesions [4]. Impaired hSPCA1 expression can lead to acantholysis, as observed in HHD patients [4].

HHD most often presents in patients in their 30s to 40s with a family history of HHD [3,5]. It affects both sexes equally and presents as erythematous plaques and vesicles with overlying crust typically located in the intertriginous areas. These areas are more commonly affected due to their moist, warm, and friction-prone environment. Burning sensation, pruritus, yellow crust, and malodor can be observed with secondary infection [3]. Heat, sweat, ultraviolet radiation, and friction exacerbates the condition, thus patients are more symptomatic in summer months. 

HHD histopathology shows suprabasilar acantholysis and negative direct immunofluorescence [3]. There is no gold standard treatment at this time. A variety of management methods with variable success are reported in the literature, including topical antibiotics, topical calcineurin inhibitors, topical and systemic corticosteroids, botulinum toxin type A and naltrexone [6]. 

There are only seven reported cases of HHD with superimposed eczema herpeticum (EH) or Kaposi's varicelliform eruption [7-9]. It is often caused by a viral infection, most frequently herpes simplex virus type 1 (HSV-1) [10-12]. EH was first described in 1887 by Moritz Kaposi. EH presents as painful vesiculopustular with erosions and multinuclear giant cells with intranuclear inclusion on histology [11]. Both histopathologic features of HHD and EH can be observed in coexisting pathology like that of our patient. EH is often diagnosed late because the eruptions are difficult to differentiate with preexisting conditions [13]. HSV infection can exacerbate HHD lesions and increase mortality and morbidity if not treated appropriately and in a timely manner [9]. We report an interesting case of recalcitrant HHD with superimposed EH caused by HSV-2 infection in a 71-year-old Caucasian female managed in a burn unit.

## Case presentation

A 71-year-old female patient with a past medical history of recalcitrant HHD, family history of HHD, hypertension, hypothyroidism, hyperlipidemia, and depression was admitted to the burn center for intractable pain secondary to scaly, blistering, erythematous, and severely disseminated HHD skin lesions. The patient was not responsive to outpatient treatment regimens including oral prednisone, dapsone, and clindamycin. The patient's admit lab results are provided in Table 1. 

**Table 1:** Laboratory results at presentation GFR: Glomerular filtration rate; CKD-EPI: Chronic kidney disease epidemiology collaboration; HSV NAT: Herpes simplex virus nucleic acid amplification test; VSV NAT: Varicella-zoster virus nucleic acid amplification test.

White Blood Cell Count	12.37 K/cu mm
Red Blood Cell Count	3.68 M/cu mm
Hemoglobin	10.1 g/dl
Hematocrit	32,8%
Mean Corpuscular Volume	89.0 fL
Mean Corpus Hgb	27.7 pg
Mean Corpus Hgb Concentration	30.8 g/dL
RBC Distribution Width	18.9%
Platelet Count	558 K/cu mm
Mean Platelet Volume	10.9 f L
Nucleated RBC Number	0.00 K/cu mm
Neutrophil %	76.3%
Lymphocytes %	13.7 %
Monocyte %	6.2 %
Eosinophil %	4.5 %
Basophil %	0.2 %
Immature Gran %	1.2 %
ANC-Neutrophil Absolute	8.10 K/cu mm
Lymphocytes absolute	1.46 K/cu mm
Monocyte absolute	0.66 K/cu mm
Eosinophil Absolute	0.32 K/cu mm
Sodium	132 mmol/L
Potassium	4.3 mmol/L
Chloride	93 mmol/L
Carbon Dioxide	31 mmol/L
Urea Nitrogen	11 mg/dL
Creatine, serum	0.53 mg/dL
Est GFR Afr-Am (CKD-EPI Eqn)	110 mL/min/1.73sqm
Est GFR nonAfram (CKD-EPI Eqn)	95 mL/min1/73sqm
Glucose	95mg/dL
Calcium	7.5 mg/dL
Total Protein	5.0 g/dL
HSV 1 NAT	Not Detected
HSV 2 NAT	Detected
VSV NAT	Not Detected

While in the burn center, the patient was started on topical triamcinolone 0.1% ointment for local wound care, 400 mg TID acyclovir for herpes prophylaxis, and 2-4 mg morphine for pain issues. Her wounds showed minimal improvement with local wound care; however, she continued to have significant pain associated with daily dressing changes. After consulting with dermatology, a novel naltrexone therapy was suggested. Oral 1.5-3 mg naltrexone treatment was initiated and maintained over two days, but it was discontinued due to drug intolerance. The patient was experiencing worsening pain, tachycardia, tachypnea, dizziness, and anxiety. It was decided to keep the patient in the hospital until her wounds had crusted over completely and showed general healing. The patient spent an additional eight days in the hospital and was discharged home with skilled nursing, physical therapy, and occupational therapy. On her clinic follow up one month later, her disseminated wounds were notably dry, crusted without any open areas. Her pain continued to be her main issue and she was referred to outpatient pain services. On her two months follow up, her pain had improved as her overall wound burden had decreased.

## Discussion

There should be high suspicion of secondary infection, especially HSV, Staphylococcus, or Streptococcus, with recalcitrant HHD with intractable pain and slow-healing wounds. HHD concomitant HSV infection is very rare and often difficult to differentiate. Delaying secondary EH diagnosis and treatment is found to increase hospital stays, which can cause further patient suffering, financial burden, and poor outcomes [14].

Managing HHD is readily complex due to its rarity and arbitrary therapy options. The complexity becomes further compounded when it comes to treating recalcitrant HHD with concomitant herpetic eczema, especially in a severe case like our patient. Viral infections exacerbate HHD lesions, which can further cause the dissemination of old and new infections [9]. Given the complexity of HHD with superimposed HSV infection, we suggest management in an inpatient burn unit to allow for proper wound care and pain management by a highly skilled multidisciplinary team. Wound care should consist of topical steroids and possibly topical antimicrobials to decrease recovery time and to control old/new infections. It is crucial that pain is controlled with multimodalities, including non-steroidal, neuromodulating and opiates for wound care. When multiple organs are involved, having rapid access to multidisciplinary care is critical for successful management. 

There is no gold standard treatment with HHD with secondary HSV-2 infection, but anti-viral - acyclovir, famiciclovir, and valacyclovir - has been the standard of care for EH [9]. It is highly suggested to treat with acyclovir prophylactically in patients with suspicious clinical presentations to avoid severe complications [7,9]. With our patient, HHD was managed with triamcinolone ointment, and EH was managed with acyclovir with a good response. It has been recommended that systemic antibiotics and topical silver sulfadiazine can be used prophylactically [10]. Our patient did not receive any oral antibiotics as no physical or laboratory bacterial infection was suspected. Rapid diagnosis of superimposed HSV infection is vital. Viral cultures, Tzanck smear, direct immunofluorescence, and polymerase chain reaction are good diagnostic options. 

Low-dose naltrexone (LDN) has been shown to decrease patients’ pain burden and to yield insignificant changes in opioid requirements [15]. LDN treatment was started but discontinued after two days of therapy due to drug intolerance. Our patient experienced severe dizziness, confusion, and anxiety during and after LDN infusion. Pregabalin was added to her pain regimen along with the aggressive treatment of HSV-2 with acyclovir. 

The use of naltrexone to treat HHD first surfaced in the literature in 2016. There are four published case series with promising results. Albers et al. reported the administration of naltrexone 3 mg to 4.5 mg for three patients (50+ years of age) and observed promising results in just two weeks, and complete resolution in just two months [16]. Ibrahim et al. reported three patients who were treated with 1.5 mg to 3 mg naltrexone with close to 80% resolution by 3-4 months of treatment [17]. Similarly, Cao et al. reported a positive response in two of the three patients who received naltrexone treatment and suggested the possibility of using 12.5 mg to 50 mg doses for adequate response [18]. In another case report, four members within the same family, all diagnosed with HHD, were treated with LDN with a good response [19]. In addition, a flare-up of HHD is common after discontinuation of naltrexone within the reviewed articles [16-19]. 

The mechanism of action of LDN on HHD is not precisely clear, but it seems to play a role in keratinocytes stabilization through inhibition of m-opioid receptors and calcium hemostasis with anti-inflammatory effects through toll-like receptor 4 antagonist leading to a paradoxical up-regulation of endogenous opioids and opioid receptors [15]. LDN therapy maybe of benefit in selected cases, but further clinical research studies are needed to make any final recommendations.

## Conclusions

We present a rare case of recalcitrant HHD with superimposed EH caused by HSV-2 infection in a 71-year-old Caucasian female managed in a burn unit. It is highly advised that such cases be managed in burn centers that offer multimodalities for prompt, rigorous management. There needs to be high suspicion of secondary infection in severe recalcitrant HHD; early diagnosis and treatment are highly suggested for EH to avoid fatal complications.
